# Deterioration of mitochondrial bioenergetics and ultrastructure impairment in skeletal muscle of a transgenic minipig model in the early stages of Huntington's disease

**DOI:** 10.1242/dmm.038737

**Published:** 2019-07-26

**Authors:** Marie Rodinova, Jana Krizova, Hana Stufkova, Bozena Bohuslavova, Georgina Askeland, Zaneta Dosoudilova, Stefan Juhas, Jana Juhasova, Zdenka Ellederova, Jiri Zeman, Lars Eide, Jan Motlik, Hana Hansikova

**Affiliations:** 1 Laboratory for Study of Mitochondrial Disorders, Department of Pediatrics and Adolescent Medicine, First Faculty of Medicine, Charles University and General University Hospital in Prague, 12108 Prague 2, Czech Republic; 2 Laboratory of Cell Regeneration and Cell Plasticity, Institute of Animal Physiology and Genetics AS CR, 27721 Liběchov, Czech Republic; 3 Department of Medical Biochemistry, University of Oslo and Oslo University Hospital, 0372 Oslo, Norway

**Keywords:** Huntington's disease, Mitochondrial function, Ultrastructure, HD large animal model, Disease development, Skeletal muscle, Biomarkers

## Abstract

Skeletal muscle wasting and atrophy is one of the more severe clinical impairments resulting from the progression of Huntington's disease (HD). Mitochondrial dysfunction may play a significant role in the etiology of HD, but the specific condition of mitochondria in muscle has not been widely studied during the development of HD. To determine the role of mitochondria in skeletal muscle during the early stages of HD, we analyzed quadriceps femoris muscle from 24-, 36-, 48- and 66-month-old transgenic minipigs that expressed the N-terminal portion of mutated human huntingtin protein (TgHD) and age-matched wild-type (WT) siblings. We found altered ultrastructure of TgHD muscle tissue and mitochondria. There was also significant reduction of activity of citrate synthase and respiratory chain complexes (RCCs) I, II and IV, decreased quantity of oligomycin-sensitivity conferring protein (OSCP) and the E2 subunit of pyruvate dehydrogenase (PDHE2), and differential expression of optic atrophy 1 protein (OPA1) and dynamin-related protein 1 (DRP1) in the skeletal muscle of TgHD minipigs. Statistical analysis identified several parameters that were dependent only on HD status and could therefore be used as potential biomarkers of disease progression. In particular, the reduction of biomarker RCCII subunit SDH30 quantity suggests that similar pathogenic mechanisms underlie disease progression in TgHD minipigs and HD patients. The perturbed biochemical phenotype was detectable in TgHD minipigs prior to the development of ultrastructural changes and locomotor impairment, which become evident at the age of 48 months. Mitochondrial disturbances may contribute to energetic depression in skeletal muscle in HD, which is in concordance with the mobility problems observed in this model.

This article has an associated First Person interview with the first author of the paper.

## Abstract (precis)

**Summary:** Specific mitochondrial parameters in muscle of a minipig model of Huntington's disease can be used as potential biomarkers of disease progression and suggest that similar pathogenic mechanisms underlie disease progression in TgHD minipigs and HD patients.

## INTRODUCTION

Huntington's disease (HD) is neurodegenerative disorder caused by the expansion of a polyglutamine stretch within the huntingtin protein (Htt) (Vonsattel and DiFiglia, 1998; Novak and Tabrizi, 2010). The disease is caused by an expansion that leads to the inclusion of over 35 CAG repeats in exon 1 of the huntingtin gene (*HTT*), resulting in the production of mutated Htt protein (mHtt) (Kremer et al., 1994). Ubiquitous mHtt expression not only causes lesions in specific brain areas but also acts in peripheral tissues, including skeletal and cardiac muscle, and blood cells (Carroll et al., 2015; Trottier et al., 1995).

Peripheral pathologies include HD-related skeletal muscle malfunction. HD patients lose weight, despite normal or even elevated food intake. The weight loss observed in both HD patients and animal models is associated with increased energy expenditure and global muscle wasting that is independent of locomotor activity (van der Burg et al., 2009). Recent studies have described progressive skeletal muscle atrophy that is demonstrated by a decline in mass in all skeletal muscles types in both fast- and slow-progressing mouse models of HD, including R6/2, *Hdh*Q150 and BACHD (Mielcarek et al., 2015; She et al., 2011; Valadão et al., 2017). Recent evidence indicates that muscle wasting in HD may occur independently of basal ganglia and cortex dysfunction (Mantovani et al., 2016; van der Burg et al., 2009).

Several studies have reported defects in mitochondrial functioning in HD patients and models that were observed not only in the central nervous system but also in peripheral tissues such as skeletal muscles (Reddy, 2014; Gizatullina et al., 2006; Hering et al., 2017). The possible role played by mitochondria in the pathogenesis of HD in muscle is supported by findings including respiratory chain complex (RCC) IV deficiency (Kosinski et al., 2007), reduced muscle strength (Busse et al., 2008), decreased ATP/phosphocreatine levels (Lodi et al., 2000) and a lower anaerobic threshold and higher lactate production after exercise (Ciammola et al., 2011) in HD patients. Furthermore, cultivated myoblasts demonstrated a higher level of glycolysis and ultrastructural impairment of mitochondrial cristae (Ciammola et al., 2011). Patient myoblasts also showed perturbations in the mitochondrial membrane potential and cytochrome *c* release and defective cell differentiation (Ciammola et al., 2006). Unfortunately, the specific roles played by mitochondria and mitochondrial energetic metabolism in skeletal muscle during the preclinical and early clinical stages of HD have not yet been investigated in detail.

The objective of this study was to monitor mitochondrial function in skeletal muscle during the development of HD. To achieve our goal, we used transgenic minipigs that contain the N-terminal portion of mutated human huntingtin protein (TgHD) (Baxa et al., 2013), which represent a large animal model of HD that shows a slow progression of HD (Askeland et al., 2018b; Vidinská et al., 2018) and is thus an appropriate representation of the human HD patient. We examined the mitochondria within the skeletal muscle of the TgHD minipigs in terms of ultrastructure, function and protein characteristics at ages between 24 and 66 months during disease progression.

## RESULTS

Fresh muscle samples collected under standard conditions at the ages of 24, 36, 48 and 66 months were used. All analyses in individual animals were performed using the same muscle sample, which allowed us to correlate all of the parameters with one another.

### Presence of mHtt in skeletal muscle of the HD model

First, we monitored the occurrence of mHtt in skeletal muscle samples from the TgHD animals. mHtt was found in TgHD muscles at all ages examined (Fig. S1). This finding suggests a possible interaction between mHtt and mitochondria in the TgHD minipig muscle at all ages. The anti-huntingtin antibody EPR5526 revealed the presence of mHtt (115-117 kDa) in TgHD samples and endogenous Htt (346 kDa) in all samples. High levels of mHtt (115-117 kDa) and fragmented mHtt (60-70 kDa) were detected using the anti-poly Q 1C2 antibody.

### Body weight and correlation with selected biochemical parameters

The body weight (BW) of the animals, which is an important factor that is correlated with muscle atrophy and cachexia in HD, was recorded on the day of the sample collection. There were no significant differences in the BW between the TgHD and WT animals or between animals of either sex at the ages of 24-66 months (Fig. S2; sex not shown). However, BW was shown to be correlated with selected mitochondrial parameters related to HD (Fig. S5). In particular, complex II (CII)- and complex IV (CIV)-dependent respirations were significantly correlated with BW (*P*=0.0003 and *P*=0.0026, respectively) and differed in the TgHD and WT animals (Table S1, Fig. S5A,B). The subunits of RCCV, F1a and OSCP, correlated significantly with BW (*P*=0.0248, *P*=0.0386, respectively) and the curves reflecting this correlation had different slopes (*P*=0.0061, *P*=0.0176) for each group (Fig. S5C,D; Table S1F). The correlation of SDH30 (subunit of RCCII) and nDNA damage with BW differed in the TgHD and WT groups (*P*=0.0401; *P*=0.0421; Fig. S5E,F).

### Skeletal muscle in TgHD minipigs showed ultrastructural abnormalities

**Fig. 1.**
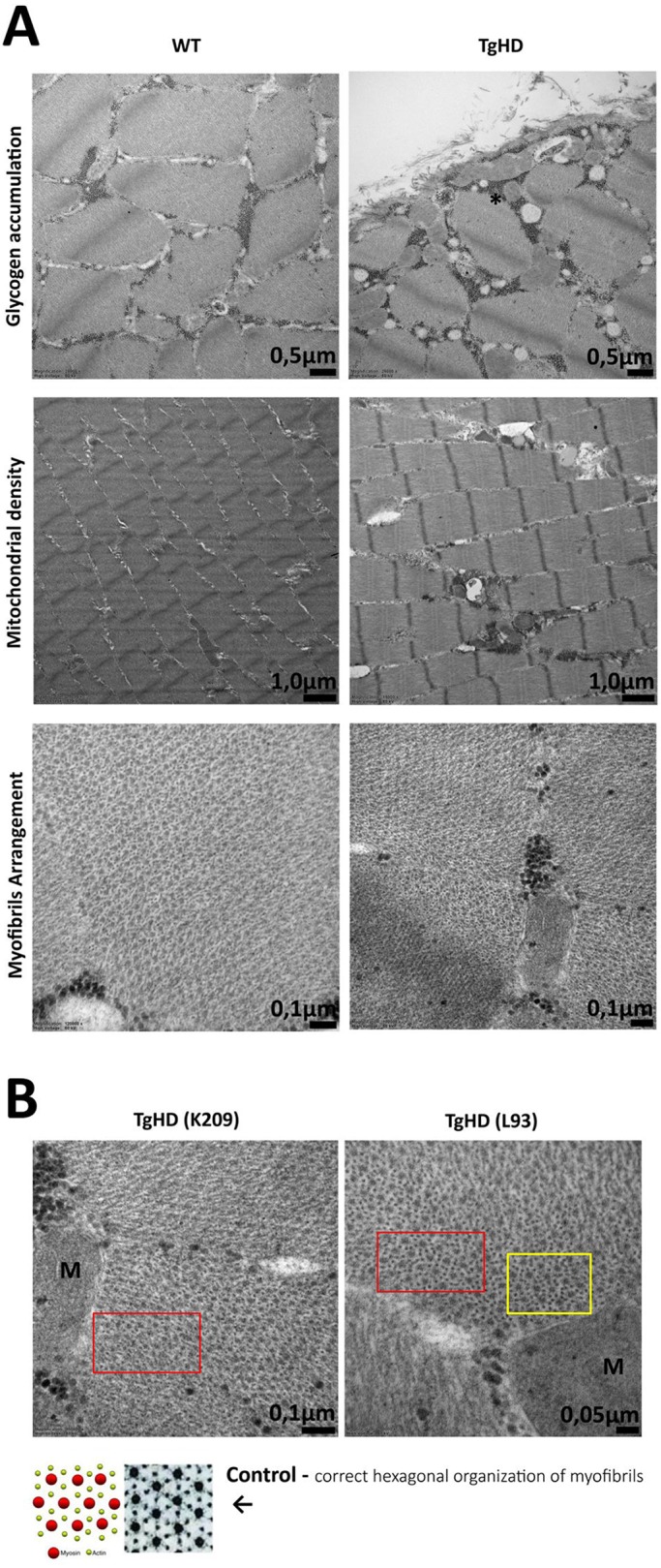
**Ultrastructure of skeletal muscle from 48-month-old WT and TgHD minipigs analyzed using transmission electron microscopy.** (A) Accumulation of glycogen, mitochondrial density and myofibril arrangement in WT and TgHD muscle. Glycogen is indicated in TgHD muscle with an asterisk (top). (B) Higher mitochondrial density and local disruption of hexagonal organization of myofibrils is observed in TgHD animals (red boxes). The amount of actin is also increased (yellow box); M, mitochondrion. The selected pair represents the results of the entire group. TgHD, transgenic; WT, wild type; K209, L93, animal identification numbers.

Ultrastructural abnormalities have been found in the mitochondria of the biceps brachii muscle in HD patients (Ciammola et al., 2006). Therefore, we investigated mitochondrial ultrastructure in minipig muscle samples using electron microscopy analyses, which revealed significant accumulation of glycogen granules and greater mitochondrial density in the muscles of TgHD minipigs (Fig. 1A). We also observed an aberrant organization of myofibrils in muscles at the age of 48 months (Fig. 1B). Similar observations were made in minipig muscles at the age of 66 months (data not shown).

### Reduced function of the respiratory chain and Krebs cycle was observed in skeletal muscle of TgHD minipigs

**Fig. 2.**
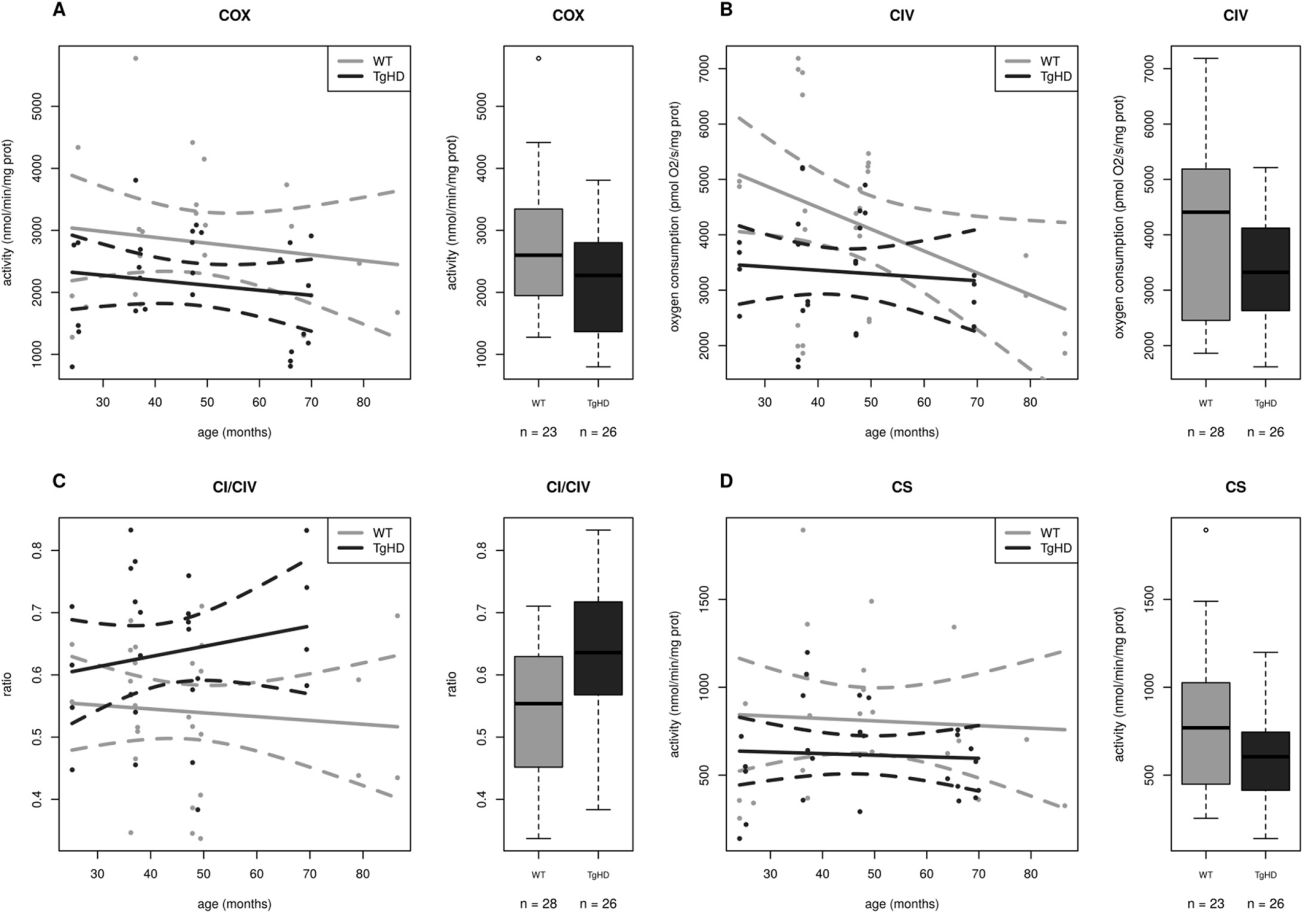
**Time course of respiratory chain complex IV and citrate synthase functional impairment in TgHD minipig skeletal muscle during the development of HD.** (A) Specific activity of the respiratory chain complex IV (COX) is significantly reduced in TgHD animals in comparison with WT animals (*P*=0.0271). (B) Complex IV (CIV)-dependent respiration was significantly decreased in TgHD animals (*P*=0.0003). (C) The ratio of respiratory parameters CI/CIV is significantly increased in the TgHD group, which may indicate reduced functionality of RCCIV in TgHD animals (*P*=0.0164). (D) The activity of citrate synthase is significantly reduced in TgHD animals (*P*=0.0171). Analyses were performed with samples collected at the ages of 24, 36, 48 and 66 months. In the box-whisker plots, median and quartiles are displayed. Circles represent outliers that are further than 1.5IQR from the corresponding quartile. Whiskers show the range of non-outliers. Boxes represent the TgHD and WT groups that consist of all animals in all ages between 24 and 66 months; numbers of analyzed samples are indicated. Enzyme activities were measured spectrophotometrically and respiration was measured by high-resolution respirometry using an OROBOROS oxygraph.

**Fig. 3.**
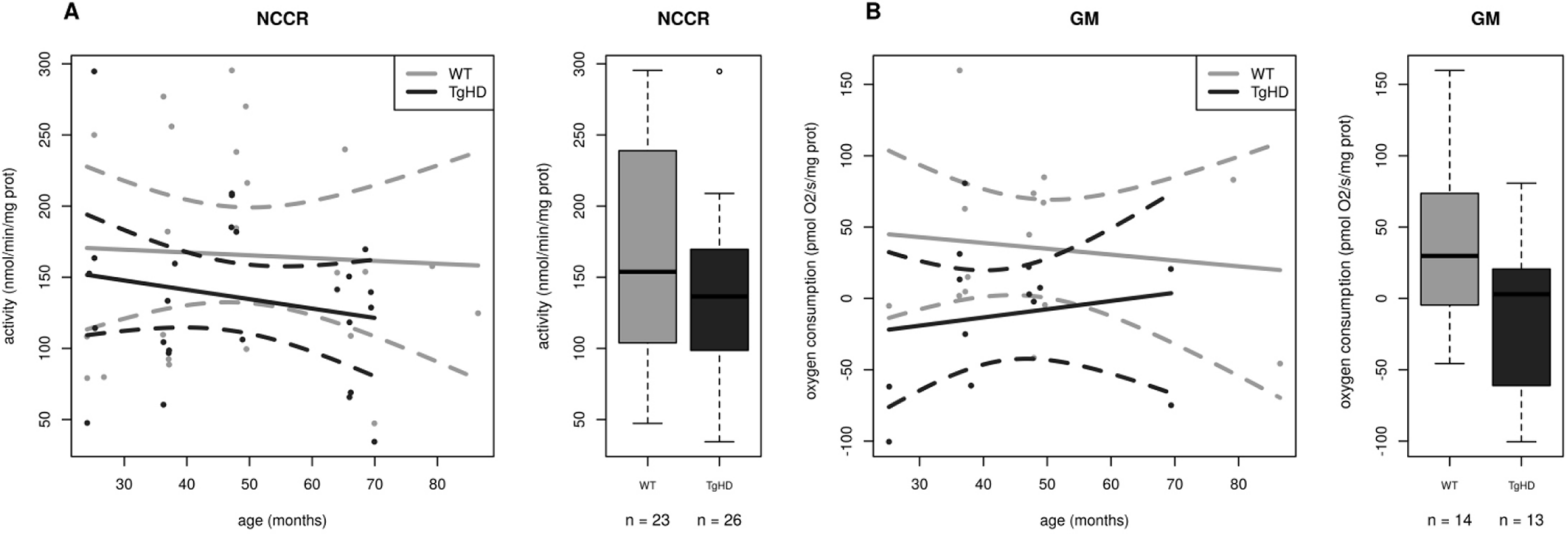
**Decreased function of respiratory chain complex I in TgHD minipig skeletal muscle during the development of HD.** (A) Activity of complex I+III (NCCR, NADH:cytochrome *c* reductase) is significantly decreased (*P*=0.0249) in TgHD muscle. (B) Complex I-dependent respiration (GM) is significantly decreased in TgHD animals (*P*=0.0321). Analyses were performed with samples collected at the ages of 24, 36, 48 and 66 months. In the box-whisker plots, median and quartiles are displayed. Circles represent outliers that are further than 1.5IQR from the corresponding quartile. Whiskers show the range of non-outliers. Boxes represent the TgHD and WT groups that consist of all animals in all ages between 24 and 66 months; numbers of analyzed samples are indicated. Enzyme activities were measured spectrophotometrically and respiration was measured by high-resolution respirometry on an OROBOROS oxygraph.

**Fig. 4.**
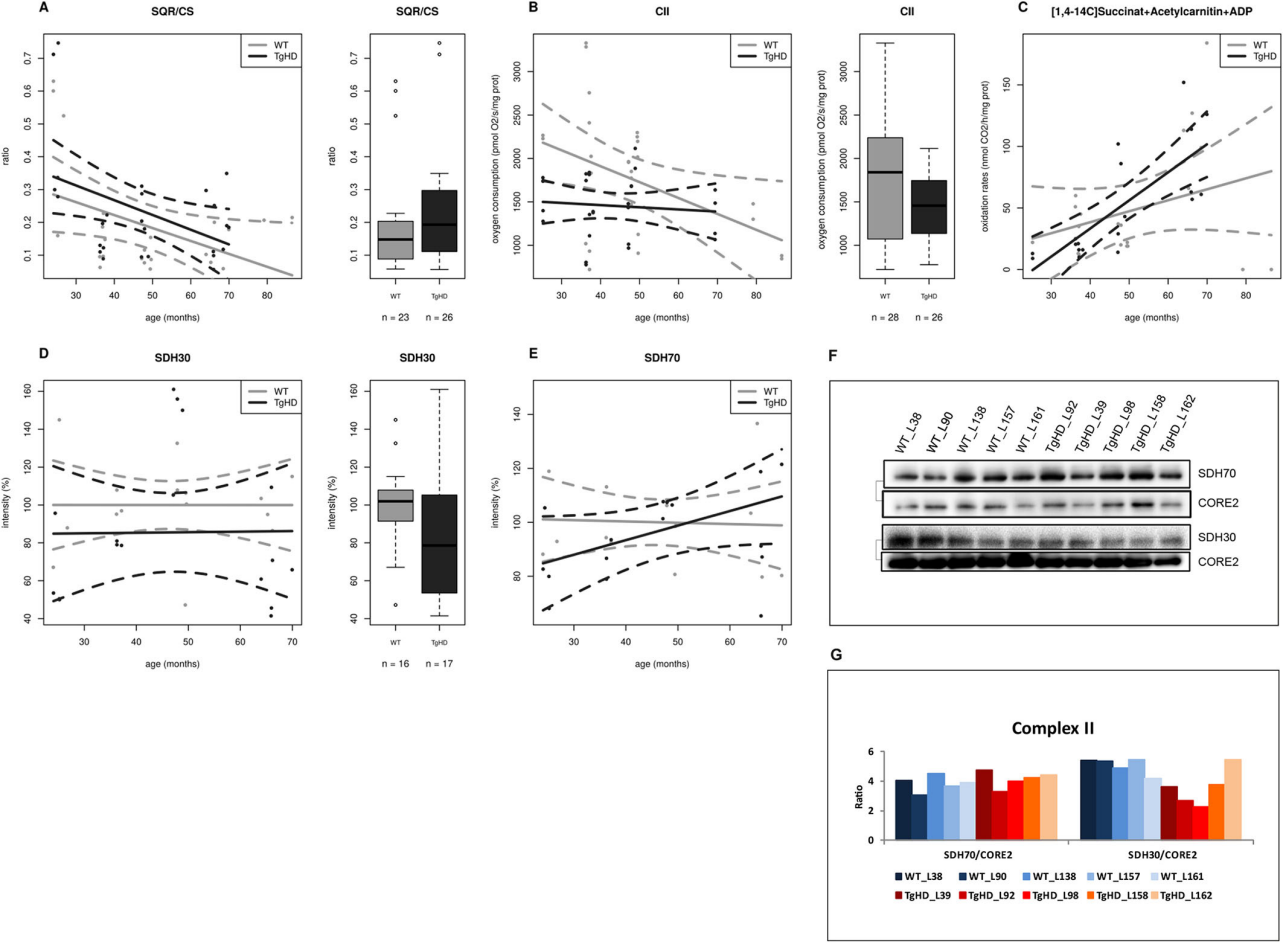
**The time course impairment of function and protein expression for complex II in TgHD minipig skeletal muscle.** (A) Ratio of complex II to citrate synthase activity (SQR/CS parameter) is significantly increased in TgHD animals compared with WT animals (*P*=0.125). (B) Complex II-dependent respiration is significantly decreased in TgHD animals (*P*=0.0008). (C) Rate of oxidation of [1,4-^14^C]succinate in TgHD animals shows an increasing trend and a different slope in comparison to WT animals, which may indicate the need for increased turnover for RCCII. (D) Expression of subunit SDH30 of RCCII is significantly lower in TgHD animals than in WT animals (*P*=0.0132). Reduced protein content was observed for all age categories. (E) In contrast, the SDH70 subunit of RCCII shows a rising trend, but the groups did not differ significantly. Intensity in panels D and E represents the signal of proteins analyzed by western blot and quantified using the Quantity One 1-D Analysis Software (Bio-Rad). In the box-whisker plots, median and quartiles are displayed. Circles represent outliers that are further than 1.5IQR from the corresponding quartile. Whiskers show the range of non-outliers. Boxes represent the TgHD and WT groups that consist of all animals in all ages between 24 and 66 months; numbers of analyzed samples are indicated. (F) Representative WB analysis of subunits SDH30 and SDH70 at the age of 66 months. The respective protein pairs from the same blot are indicated with a bracket. Analyses were conducted for samples collected at the ages of 24, 36, 48 and from 66 months. (G) Quantification of the normalized western blot signal from 5 TgHD and 5 WT muscles (shown in panel F) using Quantity One 1-D Analysis Software (Bio-Rad). Enzyme activities were measured spectrophotometrically, respiration was measured by high-resolution respirometry on an OROBOROS oxygraph and the protein content was analyzed using specific antibodies (Abcam) for western blotting.

To assess the impact of polyQ on mitochondrial function, we applied a broad methodological approach to the observations of mitochondria in fresh skeletal muscle from TgHD minipigs. First, the activity of the RCCI (NADH:ubiquinone oxidoreductase, NQR), RCCII (succinate:CoQ reductase, SQR), RCCIII (ubiquinol:cytochrome *c* oxidoreductase, QCCR), RCCIV (cytochrome *c* oxidase, COX), RCCI+III (NADH:cytochrome *c* reductase, NCCR) and RCCII+III (succinate:cytochrome *c* reductase, SCCR) as well as citrate synthase (CS) were determined spectrophotometrically in mitochondria isolated from fresh tissue. Statistical analysis showed that four activity parameters significantly depended upon HD status but not on age or sex. The COX, CS, NCCR and SQR/CS ratio are shown in Table S1A and in Fig. 2A,D, Fig. 3A and Fig. 4A, respectively. The values of the first three activities were lower in the TgHD animals than in the WT animals. The slopes of the curves show similar trends, and no breakthrough was observed at the various ages. No differences were found between the sexes.

We next analyzed mitochondrial respiration using high-resolution respirometry (Table S1B). The analysis of respiration based on the reduction of oxygen by RCCIV can reveal which oxidative phosphorylation (OXPHOS) complex has altered activity. Substrates that result in the formation of NADH (nicotinamide adenine dinucleotide, reduced), which is oxidized by RCCI or FADH2 (flavin adenine dinucleotide, reduced), which is oxidized by RCCII, were used. Because OXPHOS is highly coupled, a defect in any of the OXPHOS complexes, as well as impaired transport of metabolites and/or dysfunction of the NADH/FADH_2_ oxidoreductases inside the mitochondrial matrix, will result in decreased respiration. Respiration was assessed in isolated mitochondria in 5 different states; hence, respiration was characterized using several specific respiratory ratios (Table S1B). Correlation analyses of 11 respiration parameters showed that 5 of these parameters, GM (glutamate+malate, *P*=0.0321), CII (*P*=0.0008), CIV (*P*=0.0003), CIVu (CIV uncoupled maximal respiration with ascorbate and TMPD as electron carriers and FCCP uncoupler; *P*=0.0005) and the CI/IV ratio (*P*=0.0164), were significantly dependent only on HD status but not on age or sex (Table S1B and Fig. 3B, Fig. 4B, Fig. 2B,C).

Finally, we measured the mitochondrial energy-generating system capacity (MEGS). The determination of MEGS is a method used for complex functional analyses of mitochondrial energetics, and it was used to analyze 10 different incubations. These incubations were analyzed in terms of incubation per CS activity ratios (normalized rates), or as ratios between certain incubations according to the method used by Janssen (Janssen et al., 2006). A total of 27 MEGS parameters were statistically analyzed; 5 parameters were found to be significantly correlated with sex and 12 parameters were significantly correlated with age (Table S1E). Although a correlation between the MEGS parameters and HD status was not found, the ratio of reactions (incubations) 1/3 and 2/1 showed different trends when compared in terms of TgHD and WT (Fig. S3A,B). Incubation 3 was performed to determine the ADP (adenine dinucleotide phosphate) stimulation factor (ratio of incubation 1 to incubation 3), which reflects the coupling state of oxidation and phosphorylation in mitochondria. It appears that the coupling of mitochondria is decreased in TgHD (Fig. S3B). Ratio 2/1 in TgHD reflected steeper growth during development compared with WT. In the case of OXPHOS or ANT (adenine nucleotide translocator) deficiency, the oxidation rate in incubation 2 would show a smaller decrease than in incubation 1, which would result in an increased incubation 2 to incubation 1 ratio; therefore, a higher 2/1 ratio would indicate decreased functioning of OXPHOS or ANT in TgHD (Fig. S3A).

We quantified the total level of coenzyme Q10, since it is an important participant in the respiratory chain. However, the total coenzyme Q10 content was similar between animals in the TgHD and WT groups, and no significant correlations with age or sex were observed (data not shown).

### TgHD minipig skeletal muscle showed changes in expression of selected mitochondrial proteins

**Fig. 5.**
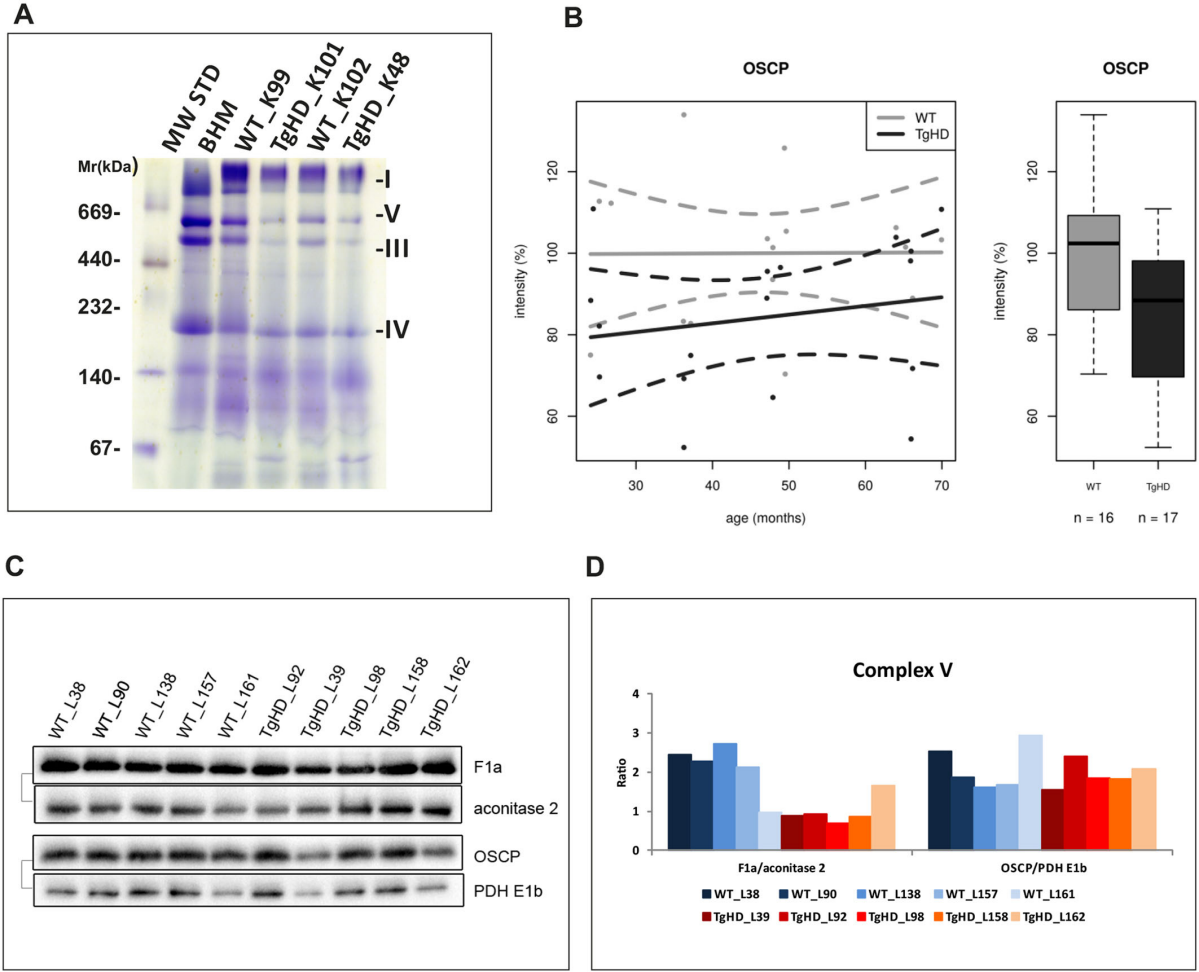
**Impairment of OXPHOS protein content in TgHD minipig skeletal muscle.** (A) Blue-native PAGE analysis of isolated skeletal muscle mitochondria from TgHD minipigs and WT controls; representative analysis at the age of 48 months is shown. Decreased amounts of RCCI, V, III and IV was found in TgHD muscle. Positions of the respiratory chain complexes are indicated on the left. (B) Decreased levels of OSCP in TgHD animals compared with WT animals (*P*=0.0176). Intensity in panel B represents the signal of proteins analyzed by western blot and quantified using the Quantity One 1-D Analysis Software (Bio-Rad). In the box-whisker plots, median and quartiles are displayed. Circles represent outliers that are further than 1.5IQR from the corresponding quartile. Whiskers show the range of non-outliers. Boxes represent the TgHD and WT groups that consist of all animals in all ages between 24 and 66 months; numbers of analyzed samples are indicated. (C) Representative western blot analysis of the OSCP and F1a subunits of RCCV at the age of 66 months; the respective protein pairs from the same blot are indicated with a bracket. (D) Quantification of normalized western blot signal from 5 TgHD and 5 WT muscles (shown in panel C) using Quantity One 1-D Analysis Software (Bio-Rad).

To investigate whether decreased mitochondrial function in TgHD minipig muscle is associated with changes in protein expression, we performed extensive immunoelectrophoretic analyses. The mitochondrial fraction was resolved using blue-native PAGE, which allows the visualization of native, intact OXPHOS complexes. Our analysis showed that the amount of mitochondrial proteins was generally reduced in TgHD samples compared with WT samples. Representative results at the age of 48 months are shown in Fig. 5A.

Next, selected OXPHOS and PDHc (pyruvate dehydrogenase complex) subunits were resolved using SDS-PAGE and then subjected to immunoblotting; signals for 17 selected proteins for each age group were statistically analyzed. Three proteins were found to be significantly dependent only on HD status: SDH30 (subunit of RCCII, SDHB, Ip), OSCP (oligomycin-sensitivity conferring protein, subunit of RCCV) and PDHE2 (subunit E2 of PDHc) (Table S1D). The SDH30 subunit content in the RCCII was significantly lower in the TgHD group (*P*=0.0132) than in the WT group, but the correlation between the protein content and HD was similar for both groups (Fig. 4D) at the observed ages. The amount of the OSCP subunit of RCCV was significantly lower (*P*=0.0357) in the TgHD group but showed a similar trend upon comparison with the WT group (Fig. 5B).

**Fig. 6.**
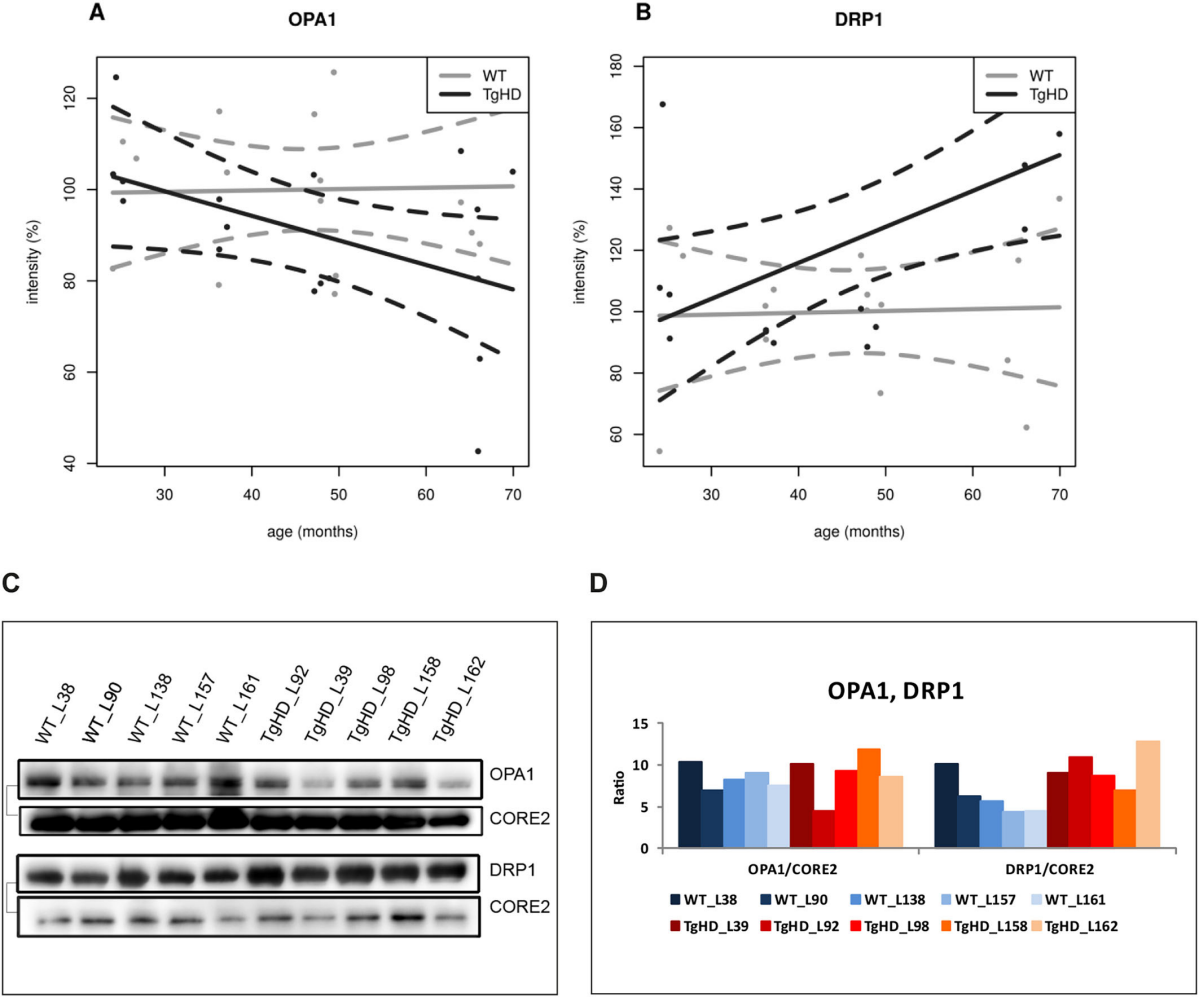
**Differential expression of selected mitochondrial membrane proteins in TgHD minipig skeletal muscle during aging.** (A) OPA1 expression showed a decreasing trend with age in TgHD animals compared with WT animals. (B) DRP1 expression showed an increasing trend in TgHD animals compared with WT animals. Western blot analyses were conducted for samples collected at the ages of 24, 36, 48 and 66 months. Intensity in panels A and B represents the signal of proteins analyzed by western blot and quantified using the Quantity One 1-D Analysis Software (Bio-Rad). (C) Representative western blot analyses of OPA1, DRP1 and CORE2 at the age of 66 months; the respective protein pairs from the same blot are indicated with a bracket. (D) Quantification of the normalized western blot signal from 5 TgHD and 5 WT muscles (shown in panel C) using Quantity One 1-D Analysis Software (Bio-Rad).

PDHE2 was significantly decreased in the TgHD group (*P*=0.0460) in comparison with the WT group and showed a decreased trend in comparison with the WT group (Fig. S6). A significant dependence on age for NDUFA9 (subunit of RCCI; *P*<0.001) and OPA1 (optic atrophy protein 1; *P*<0.001) was detected (Table S1D). Membrane proteins OPA1 and DRP1 (dynamin-related protein 1) showed interesting trends during development. Whereas OPA1 tended to reduce with age in TgHD (Fig. 6A), DRP1 increased with age in TgHD animals in comparison to WT animals (Fig. 6B).

### DNA integrity is not affected in TgHD minipig skeletal muscle

We analyzed nDNA (nuclear DNA) and mtDNA (mitochondrial DNA) damage and copy number in identical sets of muscle samples from animals aged 24-66 months. No significant differences between the DNA parameters were found in the TgHD and WT animals. However, nDNA damage in skeletal muscle was positively correlated with age (Table S1C, Fig. S4A) and mtDNA damage (*P*<0.001; Fig. S4C). The mtDNA copy number was significantly higher in females compared with males (*P*=0.02; Fig. S4E, Fig. 4F). The lack of any oxidative DNA damage was reflected by the fact that the levels of coenzyme Q10, which is an important antioxidant, were unaffected.

## DISCUSSION

### The large animal model is suitable for extensive investigations on skeletal muscle

**Fig. 7.**
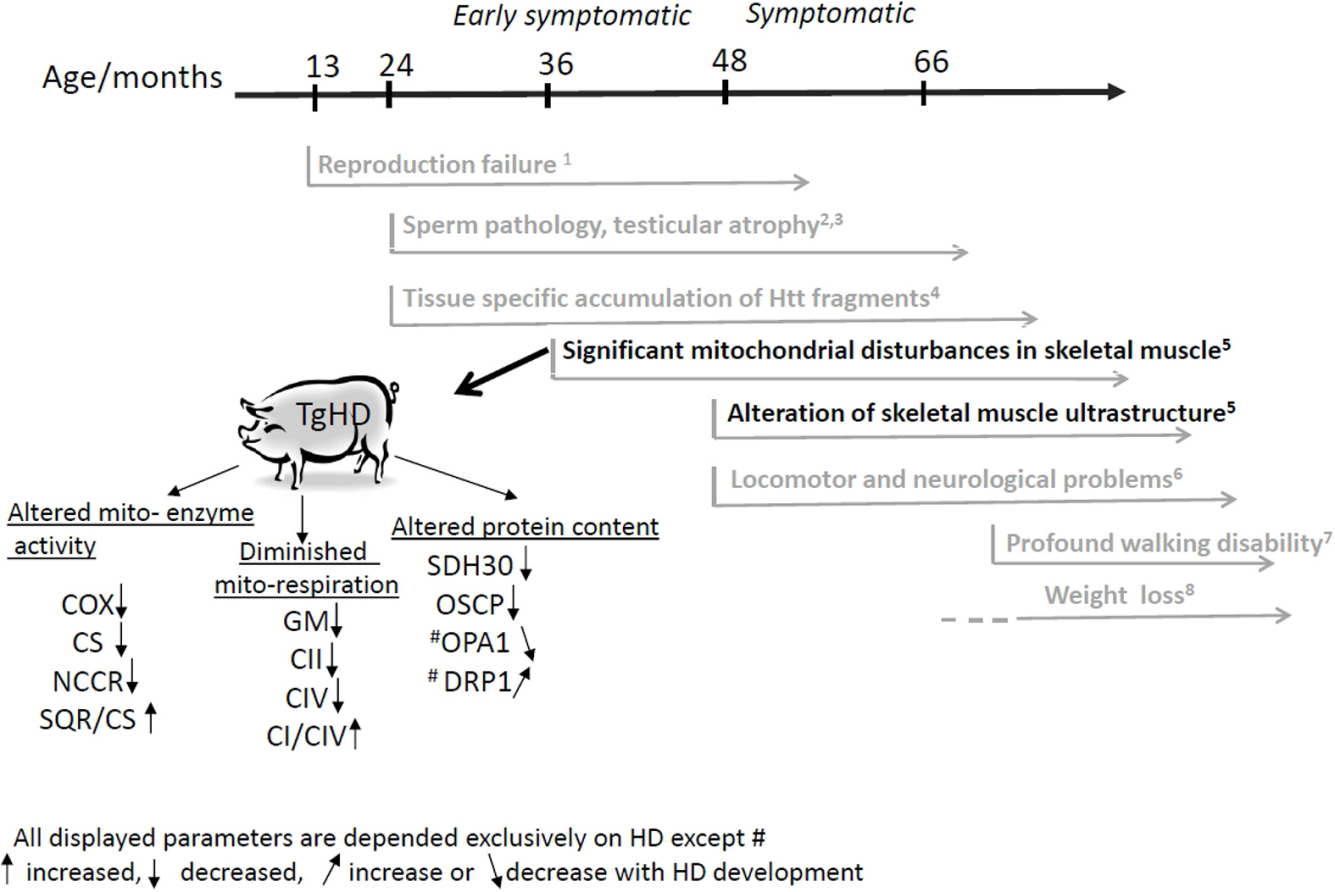
**Time-chart of HD phenotype development in TgHD minipig model.** Results from present publication are colored in black; previously published (or unpublished) data are indicted in gray. Displayed biochemical mitochondrial parameters are dependent exclusively on HD disease (except proteins marked by #) and not on age or gender, so we propose these as biomarkers of HD development. ^1^Baxa et al., 2013; ^2^Macakova et al., 2016; ^3^Krizova et al., 2017; ^4^Vidinska et al., 2018; ^5^Data described in present publication; ^6^Askeland et al., 2018b; ^7^See Movie 1; ^8^Taras Ardan, Z.E. et al., Institute of Animal Physiology and Genetics AS CR, Czech Republic, unpublished data. Enzymatic activity: COX, cytochrome *c* oxidase (RCCIV); CS, citrate synthase; NCCR, NADH-cytochrome *c* oxidase (RCCI-III); SQR/CS, ratio of succinate dehydrogenase (RCCII) to citrate synthase activity. Respiration parameters: GM, glutamate-malate (RCCI-dependent respiration); CII, RCCII-dependent respiration; CIV, RCCIV-dependent respiration; CI/CIV, ratio of RCCI- to RCCIV-dependent respiration. Proteins: SDH30, subunit of complex II; OSCP, oligomycin-sensitivity conferring protein subunit of ATPase (RCCV); OPA1, optic atrophy 1 protein; DRP1, dynamin-related protein 1.

Skeletal muscle wasting and atrophy are severe clinical impairments that are connected with the progression of HD (Zielonka et al., 2014). The existence of a large animal TgHD model allowed us to obtain sufficient tissue for our study, which is unavailable from patients owing to ethical issues. The TgHD minipig model has a slow disease progression, which is similar to that in humans. Therefore, we performed a longitudinal study focused on the mitochondrial energy metabolism in muscle, utilizing a battery of functional, ultrastructural and immunoelectrophoretic methods in a minipig TgHD model. Although we demonstrated the presence of mHtt and fragments of mHtt in muscle at all ages (Fig. S1), the animals exhibited significant locomotor functional decline from 48 months of age (Askeland et al., 2018b). Profound walking difficulties were clearly demonstrated starting from the age of 66 months (see Movie 1 at the age of 72 months and Fig. 7).

### Ultrastructural changes in minipig skeletal muscle are similar to those in HD patients

First, we analyzed the ultrastructure within the skeletal muscle samples using electron microscopy. We observed the accumulation of glycogen granules (Fig. 1) in animals that were 48 months of age and older. This age of onset is in line with the results of a previous study that identified locomotor aberrations in transgenic minipigs at 48 months (Askeland et al., 2018a). Increased glycogen production and storage was shown in isolated human skeletal muscle cells after prolonged lactate exposure (Lund et al., 2018) and elevated lactate production has also been described previously in myoblasts of HD patients (Ciammola et al., 2011). The expected increased concentrations of lactate in skeletal muscle in our TgHD model is in accordance with a demonstrated impairment of OXPHOS and PDH. We can also speculate that changes in the proportion of fiber types in TgHD and their ability to oxidize lactate in the context of their maximal pyruvate oxidative capacity may have an impact on the conversion of lactate to glycogen (Baldwin et al., 1978).

In parallel, we observed differences in the density of mitochondria in TgHD animals. Ultrastructural changes in mitochondria have also been described in the myoblasts of HD patients by Squitieri et al. (2010). These authors found rearrangement of the internal structures of mitochondria in myoblasts that consisted mostly of pure matrix loss. Myoblasts from a heterozygous HD patient with partially altered mitochondria showed dilated and spaced-out crests, and mitochondria in myoblasts from a homozygous patient displayed disruption of crests and loss of matrix content (Squitieri et al., 2010). In cross-section, normal myofibrils should create regular hexagons in muscle tissue; however, in TgHD animals, we observed disorganization of the myofibrils (Fig. 1B) and numerous impaired foci. Furthermore, it appears that the number of actin units increased. An actin rod stress response that resulted in actin remodeling has been previously described in connection with HD (Munsie et al., 2011).

### Respiratory chain complexes are significantly connected with HD

We focused on the oxidative phosphorylation system, where a significant impairment of the RCCI, RCCII and RCCIV was detected. The functional impairment of RCCIV in the TgHD minipig muscle was confirmed using several approaches (Fig. 2), which found three functional parameters for RCCIV as significant indicators for HD that could be considered as biomarkers. Decreased RCCIV activity has been previously observed in the R6/2 striatum and frontal cortex (Tabrizi et al., 2000), in R6/2 skeletal muscle in 14- to 16-week-old mice (Gizatullina et al., 2006) and in a muscle biopsy obtained from a patient many years before they developed chorea (Kosinski et al., 2007). Likewise, we confirmed the functional impairment of the RCCI in TgHD muscle (Fig. 3). The increased CI/CIV ratio (Fig. 2C) in TgHD could indicate that RCCI is less affected than RCCIV is in TgHD. Variable defects in RCCI resulting in a 25-63% reduction in activity when compared with control values have already been described in the muscles of HD patients (Arenas et al., 1998). RCCI activity was also affected in the cerebral cortex in a 3-nitropropionic acid-induced rat model of HD (Pandey et al., 2008). In contrast, a previous study of muscle biopsies from 7 pre-symptomatic patients did not show any disturbances in their respiratory parameters (Buck et al., 2017). We speculate that a defect in RCCI in skeletal muscle could be detected in parallel with the onset of functional and structural muscle cell impairment.

### Krebs cycle members play an important role in HD development

The most striking impact of HD was detected in RCCII (mainly decreased levels of subunit SDH30), which functions as part of both the respiratory chain and the Krebs cycle. Importantly, this particular subunit has also been identified as an HD biomarker in peripheral blood mononuclear cells (PBMCs) from HD patients that functions independently of age (Askeland et al., 2018a), which supports the conclusion that similar pathogenic mechanisms underlie disease progression in the TgHD minipigs and HD patients. In the spermatozoa of TgHD minipigs, we already showed that the SDH30 subunit decreases to 80% at the age of 47 months (Krizova et al., 2017). RCCII subunits are specifically decreased in the striatum of HD patients, and in Htt 171-82Q striatal neurons (Benchoua et al., 2006). RCCII subunits contain Fe-S centers that represent a specific target for superoxides. It can be predicted that damage of RCCII subunits will be observed in a number of conditions that result from oxidative stress (Rustin et al., 2002), including HD, similarly to Friedreich's ataxia (Rötig et al., 1997). Defects in the RCCII subunits would tend to reduce Krebs cycle activity and, consequently, mitochondrial ATP synthesis (Rustin et al., 2002).

In addition to impairment of RCCII functioning and protein expression, a significant decrease of the citrate synthase (CS) activity in TgHD muscle was revealed (Fig. 2D). The significantly elevated activity ratio of RCCII to CS (SQR/CS parameter) found in TgHD (Fig. 4A) may imply that CS activity is even more strongly affected than RCCII activity. CS activity is a major regulatory step that controls ﬂux throughout the Krebs cycle (Tyler, 1992). We can assume that both RCCII and CS impairment contribute greatly to decreased functioning of the Krebs cycle and that other Krebs cycle enzymes will likely be affected because of the interconnectivity of individual Krebs cycle reactions. Decreased activity of CS in ‘peripheral’ tissue has already been observed in isolated lymphocytes from HD patients (Askeland et al., 2018a), as well as in isolated thrombocytes (Silva et al., 2013).

### The mitochondrial permeability transition pore may play a role in the pathogenesis of HD

Analyses of selected membrane proteins showed that the level of oligomycin-sensitivity conferring protein (OSCP) was significantly decreased in TgHD (Fig. 5B). This parameter was dependent only on HD status and not on age or sex (Table S1D). OSCP is a subunit of the F_1_F_0_-ATP synthase (in RCCV). OSCP likely plays a fundamental role in permeability transition pore (PTP) formation and acts as a sensor for signal(s) that may induce cell death (Antoniel et al., 2014). Mitochondrial PTP (mPTP) induces mitochondrial injury in HD (Quintanilla et al., 2013). Data regarding the possible role of OSCP in neurodegeneration are insufficient, but we can assume the underlying mechanisms based on the mPTP control. Specific decreases in the levels of OSCP in the brain during the progression of Alzheimer's disease (AD) have been described in patients and an in AD mouse model (Beck et al., 2016). OSCP loss and its interplay with amyloid beta disrupts F_1_F_0_-ATP synthase, which leads to reduced ATP production, elevated oxidative stress and activated mPTP (Beck et al., 2016). More experiments should be conducted to evaluate the possible participation of OSCP in HD.

### Impaired fusion/fission apparatus in TgHD muscle may contribute to ultrastructural changes in mitochondria

Two other proteins, OPA1 and DRP1 (which are both important for the structure and function of mitochondria), showed different trends in protein expression during the development of disease in the TgHD animals (Fig. 6). OPA1 is a protein in the inner mitochondrial membrane that is responsible for maintenance of mitochondrial ultrastructure. Fusion of the mitochondrial inner membrane is regulated mainly by OPA1. In addition, OPA1 is crucial for cristae stability (Antoniel et al., 2014). DRP1 is a cytosolic GTPase that regulates mitochondrial fission, which is important for mitochondrial renewal, proliferation and redistribution (Hu et al., 2017). Excessive mitochondrial fission causes mitochondrial fragmentation, which leads to permeabilization of the outer mitochondrial membrane, ATP depletion, increase in ROS and release of apoptotic factors (Gao et al., 2017). OPA1 levels were decreased in TgHD minipig muscle compared with levels in WT minipig muscle during the progression of the disease (Fig. 6A). Our observation is supported by results found in R6/2 mice brains, where mHtt reduced the expression of *Opa1* mRNA and promoted OPA1 cleavage (Hering et al., 2017). Conversely, DRP1 levels showed an increase in TgHD minipig muscle as age increased (Fig. 6B). Overactivation of DRP1 has been shown to lead to mitochondrial dysfunction and neurodegeneration (Roe and Qi, 2018; Cherubini and Ginés, 2017) and muscle atrophy (Romanello et al., 2010) similar to that seen in HD. Therefore, we suggest that our finding of impaired fusion/fission machinery in TgHD muscle may contribute to ultrastructural changes in mitochondria.

### No changes in mtDNA of TgHD skeletal muscle

Many studies have observed changes in mtDNA resulting from HD, including decreased copy numbers and the presence of mtDNA deletions in brain tissue and leukocytes (Liu et al., 2008; Petersen et al., 2014; Banoei et al., 2007; Horton et al., 1995) in patients. In the skeletal muscle of our TgHD model, mtDNA damage was not elevated, and the mtDNA copy numbers did not differ between TgHD and WT animals. This is in concordance with our findings of impaired expression of proteins coded by nuclear DNA, rather than those encoded by mtDNA.

### Loss of body weight is not the first phenotypic manifestation in transgenic minipig model

Although at the age of 24-66 months we did not observe any decrease in body weight, which is typical of HD, we could argue that muscle mass loss will occur at an older age. This is suggested by unpublished results showing that there was a significant decrease in the animal body mass index (ABMI) in TgHD minipigs by the ages of 6 and 7 years (Taras Ardan, Z.E. et al., Institute of Animal Physiology and Genetics AS CR, Czech Republic, unpublished data). The ABMI is a more precise measured parameter in large animal models than BW, taking into account dimensions of the animal's body (Fig. 7).

### Various biochemical pathways are involved concurrently in HD pathology and development

Our 5-year study showed altered ultrastructure in TgHD muscle tissue and mitochondria, as well as significant impairment of the OXPHOS system, the Krebs cycle and the mitochondrial stabilizing membrane system (OPA1 and DRP1) in skeletal muscle of the TgHD minipig model. Owing to the close interconnection between OXPHOS and Krebs cycle, it is not clear which defect occurs first: (1) RCCII (SDH30), which results in inhibition of the Krebs cycle and ultimately the respiratory chain, or (2) decreased OXPHOS functioning, which inhibits the Krebs cycle because of disproportion in reduction equivalents or oxidative stress. Alternatively, it could be that these systems are inhibited in parallel as a result of imbalances in Ca^2+^, as previously described in HD (Panov et al., 2002). In either case, these biochemical changes precede the development of visible clinical symptoms related to muscle in TgHD minipig, when a clear correlation between the age of onset of ultrastructural changes and locomotor problems was observed. Throughout the observed period from 24 to 66 months, we did identify changes in the mitochondrial capacity to produce energy and consequent decreases in muscle functionality occurring at the age of 48 months, which was in concordance with the locomotor decline observed in the minipig model (Askeland et al., 2018b). Our study has revealed several new aspects of the mechanisms underlying HD development, such as the role of OSCP, which will be explored in our future studies.

### TgHD minipig skeletal muscle simulates the development of HD and is suitable for therapy testing focused on human medicine

The TgHD minipig represents a large animal model with a slow progression of HD (Askeland et al., 2018b; Vidinská et al., 2018) and thus is an appropriate representation of human HD. An additional value of our investigation was that our results were obtained at the end of the first third period in the minipig's lifetime (a lifetime expectancy for the TgHD minipig is 15 years), which roughly corresponds to the onset of disease in humans. We have shown that the TgHD minipig model in many mitochondrial parameters mimics the manifestations and known changes described in the muscle of HD patients (e.g. ultrastructural changes, biochemical changes of RCCI, RCCII and RCCIV, and enzymes of the Krebs cycle), which suggest that similar mechanisms underlie disease progression in this model and HD patients. In TgHD skeletal muscle, we have identified several parameters exclusively related to HD that could be used as biomarkers to track disease progression while seeking treatment and testing drugs to help improve the quality of life of HD patients with muscle symptoms, or, even better, to prevent the onset of irreversible changes in skeletal muscle. Our study has also indicated possible therapeutic targets such as mPTP. We assume that all of these data will also be beneficial to other novel large animal models with relevance to human medicine.

## MATERIALS AND METHODS

### Animals

We used transgenic minipigs that have one copy of the human *HTT* transgene, which encodes the human HD promoter and the first 548 aa, including 124 glutamines (CAG/CAA), that is integrated into chromosome 1 q24-q25 (Baxa et al., 2013). Muscle samples were obtained from minipigs from two generations (F1, F2) at the ages of 24, 36, 48 months and 66 months [total *n*=51; 27 TgHD (13 female, 14 male), 24 WT (9 female, 15 male)]; at each age, there was a minimum of 6 TgHD and 6 WT minipigs. For each experiment, the obtained TgHD samples were paired with those from the WT siblings, which were used as the paired age-related controls. The body weight of each minipig was recorded on the day of sample collection. The animals were placed under deep anesthesia and perfused with cold PBS. Fresh muscle samples from the quadriceps femoris were collected and stored at 4°C. Subsequent isolation of the mitochondria was performed within 2 h of sample collection (see below). All investigations conducted on each individual were performed using the same sample. All experiments in this study were carried out in accordance with the Animal Care and Use Committee of the Institute of Animal Physiology and Genetics and were conducted according to current Czech regulations and guidelines for animal welfare with the approval of the State Veterinary Administration of the Czech Republic.

### Homogenization and isolation of the mitochondrial fraction

A 5% homogenate (w/v) was prepared from the fresh tissue in KTEA medium containing 150 mM KCl, 50 mM Tris-HCl, 2 mM EDTA, pH 7.4 and 0.2 µg/ml aprotinin at 4°C using a Ultra-Turrax (IKA, Germany) and subsequently, a Potter–Elvehjem homogenizer (Bellco glass, Inc., USA). The postnuclear supernatant (PNS) was isolated from the homogenate by centrifugation at 600 ***g*** for 10 min at 4°C. The PNS was filtered through a nylon mesh. An aliquot of the fresh PNS was used for MEGS (mitochondrial energy-generating system capacity) analyses. The mitochondria were sedimented by centrifugation of the residual PNS at 10,000 ***g*** for 10 min at 4°C. The pellets were washed with KTEA medium, centrifuged again in the same conditions, and finally resuspended in KTEA at a protein concentration of approximately 20 mg/ml. Fresh aliquots of the isolated mitochondria were used for the enzyme activity determination and respirometry. The remaining aliquots were stored at −80°C and used for subsequent western blot (WB) analyses.

### Protein concentration

Unless otherwise indicated, the total protein concentration was determined according to the Lowry method (Lowry et al., 1951) for all analyses.

### Respirometry

Oxygen consumption in the isolated mitochondria was measured at 37°C using an OROBOROS Oxygraph-2k (OROBOROS Instruments Corp., Innsbruck, Austria) in a 2 ml chamber containing respiration medium for isolated mitochondria (pH 7.1; 0.5 mM EGTA, 3 mM MgCl_2_, 60 mM K-lactobionate, 20 mM taurine, 10 mM KH_2_PO_4_, 20 mM HEPES, 110 mM sucrose and 1 g/l BSA) (Kuznetsov et al., 2008). The protocol used multiple substrates and inhibitors, including 10 mM glutamate, 2.5 mM malate, 2.5 mM ADP+Mg^2+^, 0.5 µM rotenone, 10 mM succinate, 1 µM antimycin A, 4 mM ascorbate and 0.4 mM TMPD (N,N,N′,N′-tetramethyl-1,4-phenylenediamine) and was conducted as previously described (Kuznetsov et al., 2008). Respiration was uncoupled via titration using 200 nM steps to a maximum concentration of 1.5 μM FCCP [carbonyl cyanide p-(trifluoromethoxy) phenylhydrazone] and finally inhibited by the addition of 10 mM sodium azide. The mitochondria were verified to be intact by the addition of 2.5 µM cytochrome *c* after ADP+Mg^2+^ addition. The consumption of oxygen was expressed as pmol of O_2_ per second and was normalized based on the protein concentration in the chamber (pmol O_2_/s/mg protein). Approximately 0.05-0.1 mg protein was used for each measurement.

### Measurement of the mitochondrial energy-generating system (MEGS) capacity

The MEGS capacity in fresh postnuclear supernatant was determined by measuring the oxidation rates of [1-^14^C]pyruvate, [U-^14^C]malate or [1,4-^14^C]succinate in the presence of various donors and acceptors of acetylcoenzyme A (AcCoA) and various inhibitors of the oxidative phosphorylation system (OXPHOS) and the Krebs cycle according to the method used by Janssen (Janssen et al., 2006) using ten different incubations (Table S1E). For each reaction, 5 μl of PNS with a protein concentration of 4–8 mg/ml was used. The ^14^CO_2_ production was measured in a Beckman Coulter LS (Brea, CA, USA). The rate of each individual reaction was normalized according to both the protein concentration and the CS activity. The incubation ratios were compared to evaluate the OXPHOS activity and the functioning of the Krebs cycle and PDHc. The oxidation rates were expressed as nmol CO_2_ per minute and were normalized to the protein concentration in the reaction (nmol CO_2_/min.mg protein).

### Blue native polyacrylamide gel electrophoresis (BN-PAGE)

BN-PAGE (Schägger and von Jagow, 1991) was used for separation of the mitochondrial membrane protein complexes using 6-15% polyacrylamide (w/v) gradient gels with a Mini Protean^®^ 3 System (Bio-Rad Laboratories). Mitoplasts or mitochondria were solubilized in DDM (n-dodecyl β-D-maltoside; Sigma-Aldrich) at a final DDM/protein ratio of 1.0 mg/mg in a buffer containing 1.5 M aminocaproic acid, 2 mM EDTA and 50 mM bis-Tris (pH 7.0) at 4°C. Serva Blue G (Serva) was added to the solubilized protein at a concentration of 0.1 mg/mg detergent and 10 μg protein was loaded into each lane. Electrophoresis was performed at 40 V and 4°C for 1 h and then at 100 V and 4°C.

### Western blot analyses

The mitochondrial pellets were incubated on ice in a 2.5× volume of RIPA (radioimmunoprecipitation assay) buffer [50 mM Tris-HCl, pH 7.4, 150 mM NaCl, 1 mM PMSF (phenylmethylsulfonyl fluoride), 1 mM EDTA, 1% Triton X-100, 1% SDS (v/v)] and 1% (v/v) protease inhibitor cocktail (Sigma-Aldrich) for 20 min, with vortexing every 5 min. The microtubes were then centrifuged for 20 min at 51,000 ***g*** at 4°C, placed on ice and the supernatants were transferred to fresh tubes for determination of protein concentration (Bradford, 1976) and separation by gel electrophoresis (12% SDS-PAGE) (Fornuskova et al., 2010) using a Mini-Protean System (Bio-Rad). The protein was mixed with 4×SB (sample buffer) containing 50 mM Tris-HCl, pH 6.8, 12% (v/v) glycerol, 4% SDS, 2% (v/v) 2-mercaptoethanol and 0.01% (w/v) Bromophenol Blue for 30 min at 37°C, and 10 μg was loaded into each well of a gel along with a molecular size marker (Blue Plus2 Prestained Standard, Invitrogen). The proteins were subsequently electroblotted onto PVDF membranes (Merck). The membranes were then air-dried overnight, rinsed with 100% methanol (v/v), and blocked in TBS (Tris-buffered saline) containing 5% nonfat dried milk for 1 h. The membranes were incubated with primary antibodies in TBS containing 0.1% (v/v) Tween-20 and 2% non-fat dried milk overnight at 4°C. Secondary detection was carried out with a peroxidase-conjugated secondary antibody (Sigma-Aldrich) in TBS containing 0.1% (v/v) Tween-20 and 2% non-fat dried milk for 1 h. The proteins were visualized with the Super Signal West Femto Maximum Sensitivity Substrate (Thermo Scientific) using the Syngene Imaging System; the intensity of the signal was quantified using the Quantity One 1-D Analysis Software (Bio-Rad) during the subsequent analysis.

Primary antibodies and dilutions used, along with catalogue numbers and suppliers were: complex I subunit NDUFA9: anti-NDUFA9 antibody (20C11B11B11, ab14713, Abcam; 1:2500); complex II subunit 30 kDa IP monoclonal antibody (MS203/D1205, Mitosciences; 1:2000); complex II subunit 70 kDa Fp monoclonal antibody (MS204/D1203, Mitosciences; 1:10,000); complex III anti-ubiquinol-cytochrome *c* reductase core protein I antibody (16D10AD9AH5, ab110252, Abcam; 1:20,000); complex IV anti-MTCO1 antibody (MS404/D0804, Mitosciences; 1:6666); complex IVa anti-COX5A antibody (6E9B12D5, ab110262, Abcam; 1:4000); complex V ATP synthase subunit α monoclonal antibody (MS507/E0564, Mitosciences; 1:3000); complex V anti-ATP5O antibody (4C11C10D12, ab110276, Abcam; 1:2000); anti-mitofilin antibody (2E4AD5) – Mitochondrial Marker (ab110329, Abcam; 1:1000); purified mouse anti-OPA1 (612606, BD Transduction Laboratories; 1:2000); anti-aconitase 2 antibody (6F12BD9, ab110321, Abcam; 1:2500); anti-DRP1 antibody (ab56788, Abcam; 1:1000); anti-VDAC1/porin antibody (20B12AF2, ab14734, Abcam; 1:3000); PDH antibody cocktail (MSP02/G0351, Mitosciences; 1:2000); anti-Huntingtin (EPR5526, ab109115, Abcam; 1:1000); and poly Q antibody 1C2 (MAB1574, Merck; 1:2000). The secondary antibody used was anti-mouse IgG (whole molecule)-peroxidase antibody produced in goat (A8924, Sigma-Aldrich; 1:2500).

### Activities of mitochondrial enzymes

The activities of the mitochondrial respiratory chain complexes (RCCs) NADH:ubiquinone oxidoreductase (NQR, complex I), succinate:CoQ reductase (SQR, complex II), ubiquinol:cytochrome *c* oxidoreductase (QCCR, complex III), cytochrome *c* oxidase (COX, complex IV), NADH:cytochrome *c* reductase (NCCR, complex I+III), and succinate:cytochrome *c* reductase (SCCR, complex II+III) were measured spectrophotometrically at 37°C in freshly isolated mitochondria according to the method used by Rustin et al. (1994); citrate synthase (CS) activity was measured according to the method used by Srere (1969) using a Shimadzu 2401 UV-VIS spectrophotometer.

Briefly, to measure the activities of complexes I and I+III, approximately 20 µg of mitochondrial protein was incubated for 3 min in distilled water to disrupt the mitochondrial membranes. The rotenone-sensitive complex I activity was then measured in 1 ml of assay medium (50 mM Tris-HCl, pH 8.1, 2.5 mg/ml BSA, 50 µM decylubiquinone, 0.3 mM KCN and 0.1 mM NADH with and without 3 µM rotenone) based on the decrease in absorbance at 340 nm due to NADH oxidation (ε=6.22 mM cm^−1^).

The rotenone-sensitive complex I+III activity was determined by incubating 20 μg of mitochondria in 1 ml assay medium (50 mM Tris-HCl, pH 8.1, 2.5 mg/ml BSA, 40 mM cytochrome *c*, 2 mM KCN and 0.1 mM NADH with and without 3 µM rotenone) and measuring the increase in absorbance at 550 nm (ε=19.6 mM cm^−1^) due to reduction of cytochrome *c*.

Complex II activity (succinate-DCPIP oxidoreductase) was determined by incubating 20 μg of mitochondrial extract in 1 ml of assay medium (10 mM potassium phosphate, pH 7.8, 2 mM EDTA, 1 mg/ml BSA, 0.3 mM KCN, 10 mM succinate, 3 µM rotenone, 0.2 mM ATP, 80 µM DCPIP, 1 µM antimycin and 50 µM decylubiquinone) and measuring the decrease in absorbance at 600 nm due to the reduction of DCPIP (ε=20.1 mM cm^−1^).

Complex II+III activity was determined by incubating 20 μg mitochondria in 1 ml assay medium (50 mM potassium phosphate, pH 7.8, 2 mM EDTA, 1 mg/ml BSA, 0.3 mM KCN, 10 mM succinate, 3 µM rotenone, 0.2 mM ATP and 40 µM cytochrome *c*) and measuring the increase in absorbance at 550 nm (ε=19.6 mM cm^−1^).

Complex III activity was determined by incubating 20 μg mitochondria in 1 ml assay medium (50 mM KPi, pH 7.8, 2 mM EDTA, 1 mg/ml BSA, 0.3 mM KCN, 50 µM cytochrome *c* and 50 µM ubiquinol) and measuring the increase in absorbance at 550 nm (ε=19.6 mM cm^−1^).

Complex IV activity was determined in isolated mitochondria by incubating 20 µg of mitochondrial protein in 1 ml of assay medium (40 mM potassium phosphate, pH 7.0, 1 mg/ml BSA, 25 µM reduced cytochrome *c*, and 2.5 mM n-dodecyl-β-D-maltoside) and measuring the oxidation of reduced cytochrome *c* (II) at 550 nm (ε=19.6 mM cm^−1^).

CS activity was determined using a mixture containing 100 mM Tris-HCl, pH 8.1, 0.1 mM DTNB [5,5′-dithio-bis(2-nitrobenzoic acid)], 2.5 mM n-dodecyl-β-D-maltoside), 20 µg mitochondrial protein, 0.5 mM acetyl coenzyme A and 0.5 mM oxaloacetate. The activity was measured at 412 nm (ε=13.6 mM cm^−1^). For calculation of the final background activity, the activity without oxaloacetate was subtracted.

The activities were expressed as nmol substrate converted per minute and normalized to the protein content in the reaction (nmol/min.mg protein).

The pyruvate dehydrogenase activity was determined by measuring the ^14^CO_2_ produced by the decarboxylation of [1-14C]pyruvate in an assay containing 20-50 µg mitochondrial protein, as previously described (Cahova et al., 2015).

### Total coenzyme Q10 content

The total Q10 content in muscle homogenate was determined using HPLC with UV detection at 275 nm according to the method of Mosca et al. (2002). The results were expressed as pmol Q10/mg protein.

### Electron microscopy

Skeletal muscle was fixed in 10% paraformaldehyde at 4°C for 1 week, washed with PBS and dehydrated using an ethanol series. The dehydrated samples were embedded in Durcupan Epon, sectioned using a Ultracut Reichert microtome at a thickness ranging from 60 to 90 nm, stained with lead citrate and uranyl acetate, and observed with a Jeol JEM 1400+ transmission electron microscope.

### DNA analyses

DNA was isolated from minipig muscle samples using the DNeasy Blood & Tissue Kit (Qiagen) according to the manufacturer's protocol with minor modifications. Samples at ∼5 mm were homogenized with 1.4 mm white ceramic beads in buffer ATL for 30 s at 6.5 m/s using a Fastprep 24^®^ instrument. The samples were subsequently incubated overnight at 56°C with proteinase K and agitated to facilitate lysis. DNA concentration and purity were measured using a spectrophotometer (Epoch microplate spectrophotometer) and the samples were subsequently adjusted to the desired concentration. DNA integrity was assessed using a qPCR-based method (RADF) as previously described (Wang et al., 2016; Askeland et al., 2018a). The primers used to assess mtDNA damage were Fwd (5′-3′) TCGCAACTGCCTAAAACTCA and Rev (5′-3′) GAATTGGCAAGGGTTGGTAA. The primers used to assess nDNA damage were Fwd (5′-3′) GTTGTGAATGGTGCTAACTGCT and Rev (5′-3′) ACCAGAGACAATAAAGCAGAGGAG. The mtDNA copy number was determined using qPCR with primers for the mtDNA gene MT-RNR1 and the single copy nDNA gene (HBB), which yielded the relative mtDNA content based on the ratio of the mtDNA gene product to that of the nDNA gene using standard curves. The primers used to determine the mtDNA copy number were MT-RNR1 Fwd (5′-3′) TCGCAACTGCCTAAAACTCA and Rev (5′-3′) GAATTGGCAAGGGTTGGTAA; those used to measure the HBB gene were Fwd (5′-3′) CTCCTGGGCAACGTGATAGT and Rev (5′-3′) GGTTCAGAGGAAAAAGGGCTCCTCCT.

### Statistics

Using our set of minipig muscle samples, we focused on the statistical analysis of three possible interfering factors: (1) the effect of HD itself, (2) the effect of sex and (3) the effect of aging. The differences between the observed mitochondrial characteristics in the TgHD minipigs and the WT controls with respect to age and sex were examined using linear regression models with mixed effects. The pairs of WT and corresponding age-paired TgHD were considered as a random effect. Box-Cox transformations were used to gain the normality of residuals. For each model, the statistical significance of the parameter was tested. If the parameter was not significant at the 5% level, it was removed from the model, and a reduced model was presented. In the box-whisker plots, median and quartiles are displayed. Circles represent outliers that are further than 1.5IQR from the corresponding quartile. Whiskers show the range of non-outliers. The analysis was performed using the statistical package R version 3.4.4 (R Core Team, 2018).

## Supplementary Material

Supplementary information
