# Supplementary material for: Deterioration of mitochondrial bioenergetics and ultrastructure impairment in skeletal muscle of a transgenic minipig model in the early stages of Huntington's disease
Source: Dis Model Mech. 2019 Jul 26;12(7):dmm038737. doi: 10.1242/dmm.038737 (PMC6679385; doi:10.1242/dmm.038737)
Supplement: Supplementary information [file dmm-12-038737-s1.pdf]

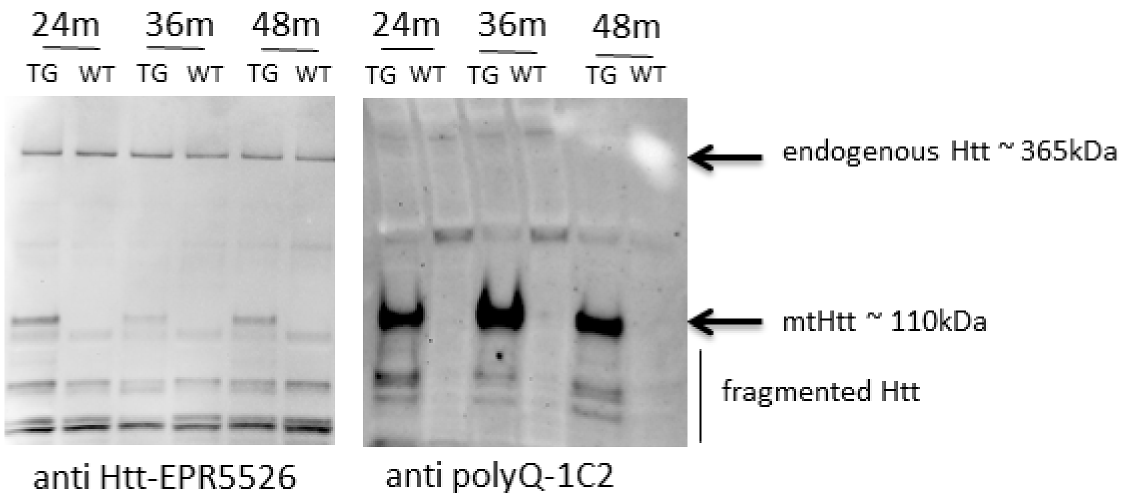

Figure S1. Presence of mHtt and mHtt fragments in skeletal muscle from TgHD minipigs at different ages during HD development. Western blot of a representative sample of each age is presented. Anti–HTT antibody EPR5526 and poly Q antibody 1C2 was used.

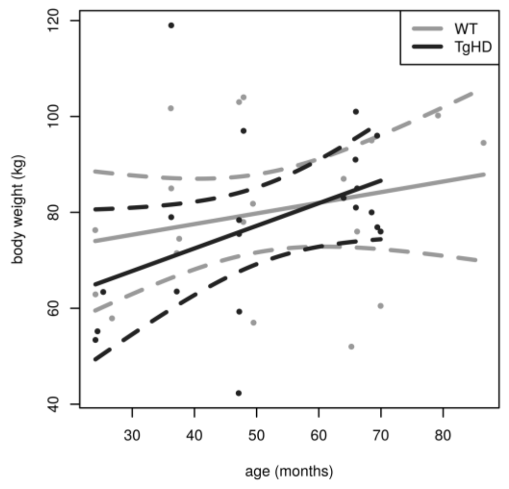

Figure S2. Body weight of animals at the time of sample collection. Body weight did not show any correlation with gender or HD status (gender not displayed).

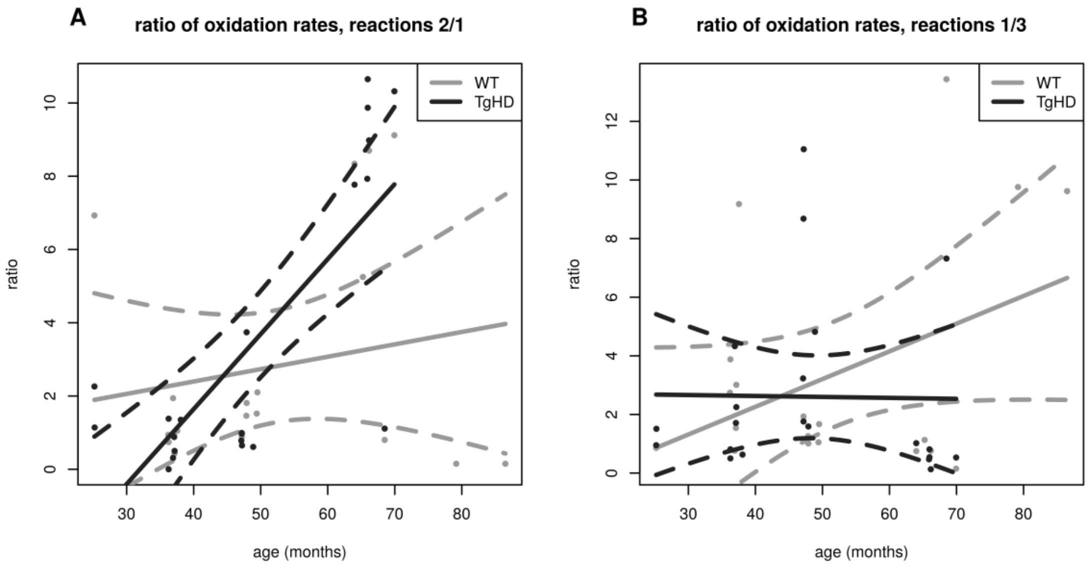

Figure S3. Mitochondrial energy-generating system capacity comparison between TgHD and WT skeletal muscle mitochondria.

Ratio of oxidation rates demonstrating the time-course progression of OXPHOS functional impairment. A. ratio of reaction 2 ([1-^{14}C]pyruvate+carnitine+ADP) to reaction 1 ([1-^{14}C]pyruvate+malate+ADP); a higher 2/1 ratio indicates decreased functioning of OXPHOS; B. ratio of reaction 1 ([1-^{14}C]pyruvate+malate+ADP) to reaction 3 ([1-^{14}C]pyruvate+malate(–ADP); incubation 3 was performed to determine the ADP stimulation factor, and ratio 1/3 reflected the coupling state for oxidation and phosphorylation in mitochondria. For both parameters, it appears that significant changes occurred at the age of approximately 48 months. Analyses were performed for postnuclear supernatants. For the technical details, see Materials and Methods.

Disease Models & Mechanisms • Supplementary information

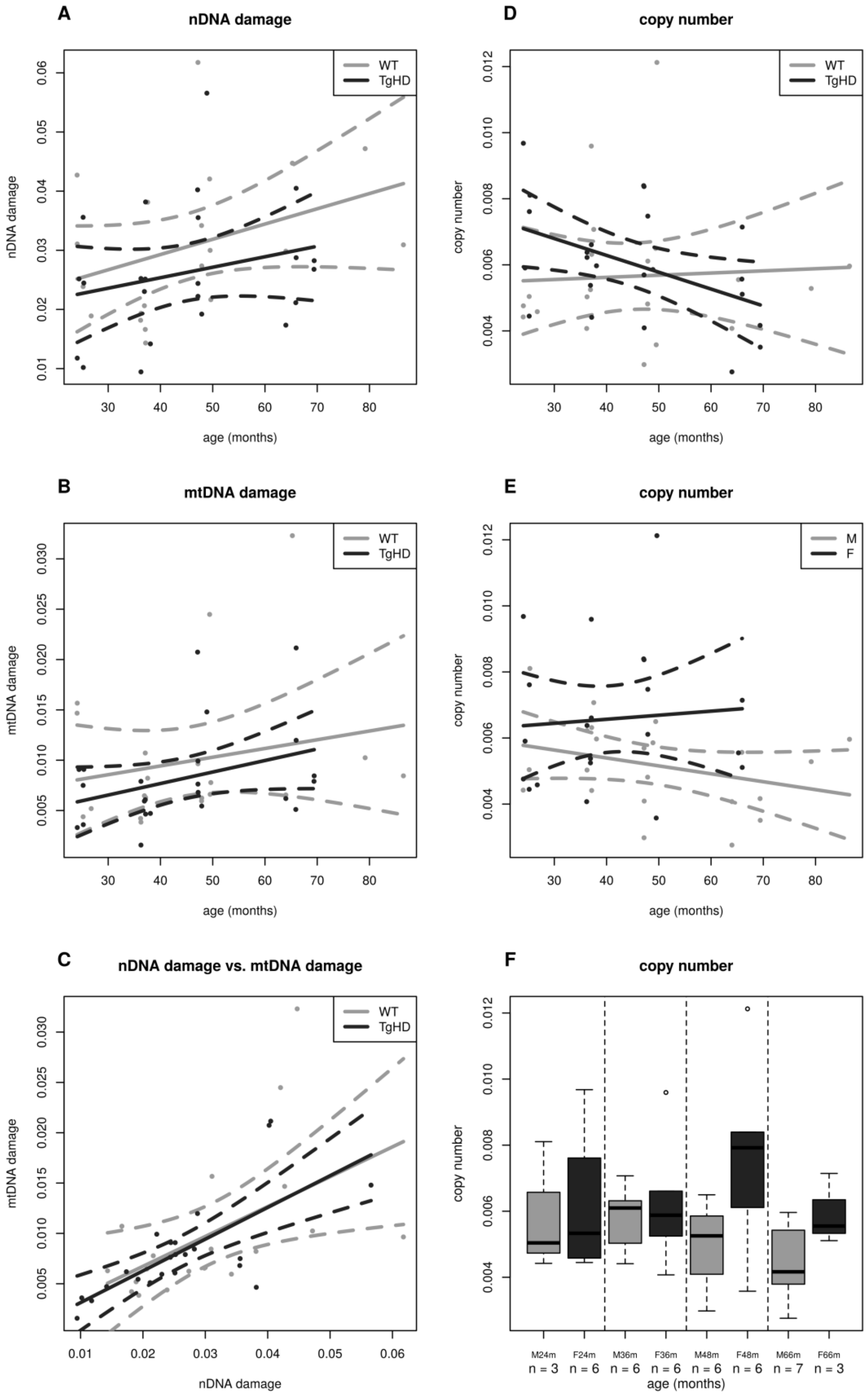

Figure S4. DNA stability and copy number analyses. A. No correlations between nDNA damage (A) or mtDNA damage (B) and HD status and gender were found. C. nDNA damage was significantly correlated with mtDNA damage (p=0.0000); D. mtDNA copy number did not differ between the HD and WT groups; E. mtDNA copy number was significantly higher in females than in males (p=0.0225); F. comparison of mtDNA copy number based on age.

Disease Models & Mechanisms • Supplementary information

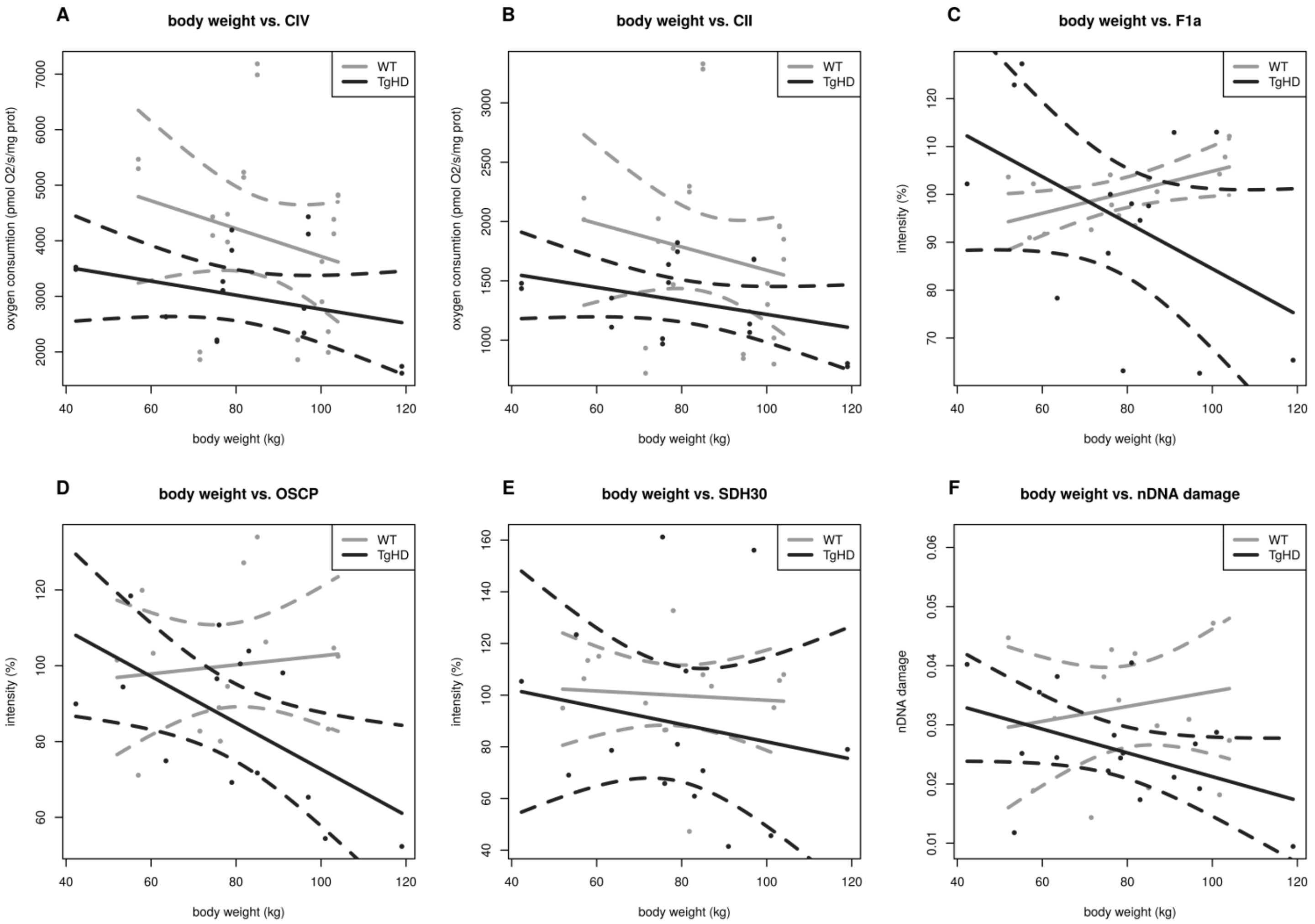

Figure S5. Correlations between body weight (BW) and selected mitochondrial parameters analyzed in skeletal muscle of TgHD and WT minipigs. A significant correlation was found for respiration parameter CIV (p=0.003) (**A**); Respiration parameter CII (p=0.0026) (**B**); content of F1a protein (p=0.0248) (**D**); OSCP (p=0.0386) (**E**). Differences between TgHD and WT animals were found for CIV (p=0.0001) (**A**), CII (p=0.001) (**B**), F1a (p=0.0061) (**C**), OSCP (p=0.0176) (**D**), SDH 30 (p=0.0401) (**E**) and nDNA damage (p=0.0421) (**F**). Intensity in panels C, D and E represents the signal of proteins analyzed by western blot and quantified using the Quantity One 1-D Analysis Software (Bio-Rad).

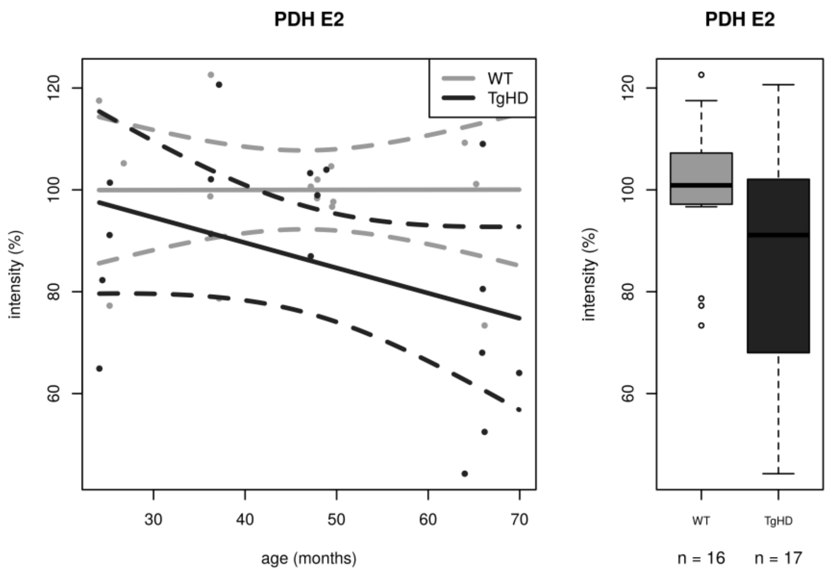

Figure S6. Significantly decreased content of the PDH E2 subunit of the pyruvate dehydrogenase complex in TgHD animals in comparison with WT. (p=0.0460) Intensity represents the signal of proteins analyzed by western blot and quantified using the Quantity One 1-D Analysis Software (Bio-Rad). In the box-whiskers plot, median and quartiles are displayed. Circles represent outliers that are more than 1.5IQR far from the corresponding quartile. Whiskers show the range of non-outliers. Boxes represent the TgHD and WT groups that consist of all animals in all ages within 24-66 month old, numbers of analyzed samples are indicated.

Table S1. p-values for all validated effects (HD, generation, age) for spectrophotometric parameters (A), respirometric parameters (B), DNA stability tests (C), protein expression parameters (D) and MEGS parameters (E). Differences between observed mitochondrial characteristics in TgHD animals and WT controls with respect to age and gender were analyzed using linear regression models with mixed effects. The pairs consisting of WT and corresponding age-paired TgHD samples were considered as a random effect. Box-Cox transformations were used to gain the normality of the residuals. For each model, the statistical significance of the parameter was tested. If it was not significant at the 5% level, it was removed from the model and a reduced model was presented. The analysis was performed in the statistical software package R (version 3.4.4).

parameter		***effect***	
A	**HD**	**age**	**gender**
enzyme activity and Q10 content			
COX	0.0121	-	-
CS	0.0278	-	-
SCCR	-	-	-
NCCR	0.0317	-	-
NQR	-	-	-
SQR	-	0.0131	-
QCCRr	-	-	-
Q10	-	0.0067	-
PDHc	-	0.0314	-
activity/CS			
COX/CS	-	-	-
SCCR/CS	-	-	-
SQR/CS	0.0125	-	-
NCCR/CS	-	-	-
B	**HD**	**age**	**gender**
respiration parameters			
GM	0.0321	-	-
PM	-	-	-
GMD	-	-	-
PMD	-	-	-
CI (GMDc-rot)	-	-	-
CII (S-Ama)	0.0008	-	-

CIV (aT-Z)	0.0003	-	-
CIVu (CIV uncoupled)	0.0005	-	-
respiratory ratios			
CI/CII	-	-	0.0073
CI/CIV	0.0164	-	-
CII/CIV	-	-	-

C	HD	age	gender
DNA integrity			
nDNA damage	-	0.0367	-
mtDNA damage	-	-	-
copy number	-	-	0.0225

D	HD	age	gender
protein content			
NDUFA9	0.0927	0.0000	-
SDH70	-	-	-
SDH30	0.0132	-	-
CORE1	0.3067	0.3933	0.0069
COX1	-	-	-
COX5a	-	-	0.0321
F1a	-	-	-
OSCP	0.0357	-	-
mitofilin	-	-	-
OPA1	0.0870	0.0000	-
PDH E2	0.0460	0.3183	0.0979
PDH E2/E3bp	-	-	-
PDH E1a	-	-	-
PDH E1b	0.3473	0.5503	0.3314
aconitase 2	-	-	-
VDAC1	-	-	-
DRP1	-	-	-
SDH30/SDH70			

E	HD	age	gender
MEGS			
(1) [1-^{14}C] Pyruvate+Malate	-	-	-
(2) [1-^{14}C]Pyruvate+Carnitine	-	0.0100	-
(3) [1-^{14}C]Pyruvate+Malate(-ADP)	-	-	0.0493
(4)[1-^{14}C]Pyruvate+Malate(-ADP)+CCCP	-	-	0.0406
(4a) [1-^{14}C]Pyruvate-Carnitine	-	-	0.0450
(5) [1-^{14}C]Pyruvate+Malate+ADP+atractyloside	-	-	0.0073
(6) [U-^{14}C]Malate+Pyruvate+Malonate	-	0.0004	-

Disease Models & Mechanisms • Supplementary information

(7) [U-^{14}C]Malate+Acetylcarnitine+Malonate+ADP	-	-	-
(8) [U-^{14}C]Malate+Acetylcarnitine+Arsenite	-	0.0152	-
(9) [1,4-^{14}C]Succinate+Acetylcarnitine	-	-	-
enzyme activity			
cs (postnuclear supernatant)	-	0.0153	-
MEGS/CS			
1/CS	-	0.0180	-
2/CS	-	0.0019	-
3/CS	-	0.0230	-
4/CS	-	-	-
4a/CS	-	0.0027	-
5/CS	-	0.0486	0.0072
6/CS	-	0.0002	-
7/CS	-	-	-
8/CS	-	0.0008	-
9/CS			
MEGS ratios	-	-	--
(1/3)	-	-	-
(2/1)	-	-	-
(4/1)	-	-	-
(3/5)	-	-	-
(6/1)	-	-	-
(7/6)	-	0.0016	-
(7/8)	-	-	-

**F**	**association**	**HD**
specific associations		
nDNA damage vs. mtDNA damage	0.0000	0.5001
body weight vs. CIV	0.0003	0.0001
body weight vs. CII	0.0026	0.0010
body weight vs. F1a	0.0248	0.0061
body weight vs. OSCP	0.0386	0.0176

* $p < 0.05$ was considered as statistically significant. Dash marks (–) indicate non-significant effects.

Activity of respiratory chain enzyme and citrate synthase (CS) were measured spectrophotometrically in freshly isolated mitochondria. NQR- complex I, NADH: ubiquinone oxidoreductase; SQR, complex II, succinate:CoQ reductase; QCCR - complex III ubiquinol:cytochrome c oxidoreductase; COX - complex IV, cytochrome c oxidase; NCCR - complex I+III, NADH:cytochrome c reductase; SCCR - complex II+III, succinate:cytochrome c reductase

Disease Models & Mechanisms • Supplementary information

Total coenzyme Q10 content was determined by HPLC in muscle homogenate. Pyruvate dehydrogenase complex (PDH) activity was determined by decarboxylation of 1-^{14}C-pyruvate in isolated mitochondria.

Respiratory states measured in freshly isolated mitochondria in medium for isolated mitochondria with: GM-rot, glutamate + malate (background corrected by rotenone inhibition); complex I-dependent respiration: GMDc-rot (CI), glutamate + malate + ADP + cytochrome c (corrected background by rotenone inhibition); complex II-dependent respiration: S-AmA (CII), succinate with glutamate + malate + ADP + cytochrome c and rotenone present in the chamber (corrected background by antimycin A inhibition); aT-Z, (CIV) maximal respiration with ascorbate and TMPD as electron carriers (autoxidation background corrected by sodium azide); aTF-Z (CIVu), CIV uncoupled maximal respiration with ascorbate and TMPD as electron carriers and FCCP uncoupler (autoxidation background corrected by sodium azide). G - glutamate, M - malate, D – ADP

Protein content was analyzed by western blot, the WB signal was quantified using Quantity One 1-D Analysis Software (Bio-Rad).

DNA integrity was determined by qPCR. nDNA - nuclear DNA, mtDNA - mitochondrial DNA

MEGS analysis measure production of $^{14}CO_2$ in postnuclear supernatant formed by oxidation of radiolabeled substrate in the incubation containing specified substrates. All MEGS incubations were supplemented with ADP unless stated as –ADP. HD - Huntington's disease, MEGS - mitochondrial energy-generating system capacity, CS - citrate synthase activity.

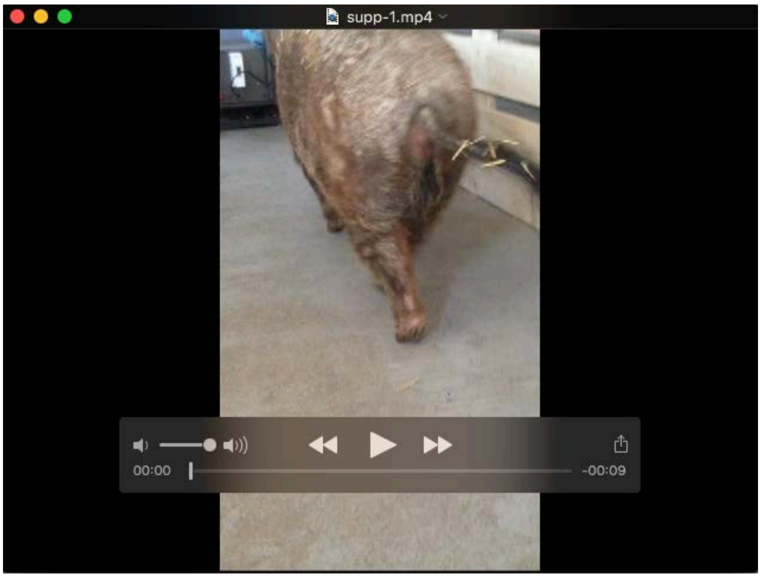

Movie 1. Demonstration of walking problems in TgHD minipigs at the age of 72 months.
